# Scheimpflug imaging of pediatric posterior capsule rupture

**DOI:** 10.4103/0301-4738.49404

**Published:** 2009

**Authors:** Dilraj Singh Grewal, Rajeev Jain, Gagandeep Singh Brar, Satinder Pal Singh Grewal

**Affiliations:** Grewal Eye Institute, Sector 9-C, Madhya Marg, Chandigarh - 160 009, India

**Keywords:** Closed globe injury, posterior capsule tear, posterior lenticonus, scheimplfug imaging

## Abstract

We report a case of an 11-year-old boy who presented two days after blunt trauma to the left eye with a slingshot. On examination his best corrected visual acuity (BCVA) was 20/20 in the right eye and 20/400 in the left eye. Slit-lamp examination of the left eye revealed a Vossius ring, traumatic cataract, traumatic posterior capsule tear (PCT). The contour of the posterior capsule bulge corresponded to the edges of the PCT. Rotating Scheimpflug imaging (Pentacam 70700:Oculus, Wetzlar Germany) confirmed the traumatic cataract in the region of the PCT visualized as increased lens density at the cortex-vitreous interface. The extent of the PCT in the greatest and least dimensions was documented before and after intraocular lens (IOL) implantation. Intra-operatively, the PCT was evident and phaco-emulsification with an IOL implant was performed. Postoperatively, his BCVA improved to 20/20 in the left eye with a well-centered in-the-bag IOL as found on slit-lamp and Scheimpflug images.

Posterior capsule tear (PCT) and cataract formation may occur following non-penetrating ocular injury.[1] Management of such cases depends to a great extent on the accurate assessment of the tear. We report a case of isolated pediatric posterior capsule tear following closed globe injury and highlight the use of Scheimpflug imaging, to visualize and quantify the size of PCT.

## Case Report

**Figure 1:**
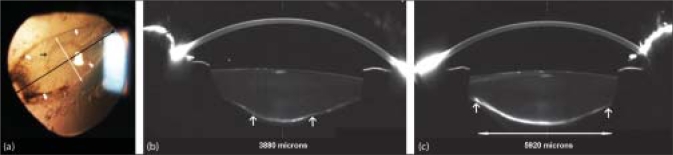
(a) Slit-lamp Photographs (SLP): slit-lamp-examination of the left-eye revealed a vossius ring (black arrow), traumatic cataract, traumatic posterior capsular tear (PCT) (thick arrow) with a bulging-out of lens cortex and a streak of blood at its lower edge (thin arrow) (b) scheimpflug images of rupture (white arrows) along the least dimensions (corresponds to white arrow on SLP). The increased density along the axis of the PCT is evident. (c): Along its greatest dimensions (white arrows correspond to black arrow along the long axis of the tear on SLP)

**Figure 2:**
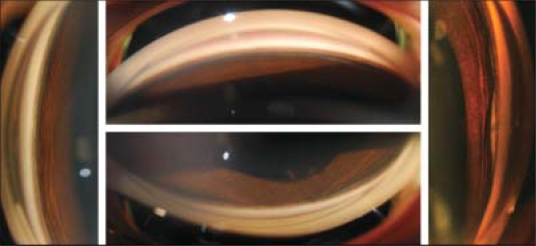
Preoperative gonioscopy pictures showing a 360-degree angle recession

**Figure 3:**
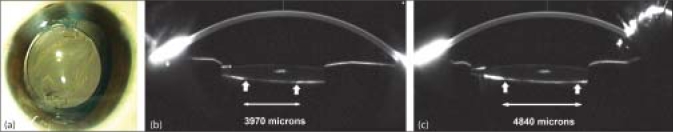
(a) Well-centered intraocular lens (IOL) post-surgery, (b) postoperative scheimpflug image at four-weeks follow-up showing a well-centered IOL and the margins of the PCT along the short axis, (c) margins of the PCT along the long axis

An 11-year-old boy presented two days following blunt trauma to his left eye caused due to a projectile released from a slingshot. On examination, his best corrected visual acuity (BCVA) was 20/20 in the right eye and 20/400 in the left eye. Slit-lamp biomicroscopy of his left eye revealed a Vossius ring, traumatic cataract, traumatic PCT with a bulging-out of the lens cortex and a streak of blood at its lower edge [Fig. 1a]. Gonioscopy revealed a 360-degree angle recession [Fig. 2]. The injury was classified as closed globe injury, Type B, Grade 3, Zone 3, relative afferent pupillary defect (RAPD) negative according to the classification of the ocular trauma classification group.[2] The contour of the posterior bulge corresponded to the edges of the PCT. Rotating Scheimpflug imaging (Pentacam 70700:Oculus, Wetzlar Germany) was performed and the images confirmed traumatic cataract in the region of PCT demonstrated as increased lens density at the cortex-vitreous interface [Fig. 1b and 1c]. The rotating Scheimpflug camera captured 50 image slices in a 360 degree circle which allowed the two dimensions of the tear to be visualized and measured. The extent of the PCT, in its least [Fig. 1b] and greatest dimensions [Fig. 1c] was documented using the linear measurement tool on Scheimpflug images prior to and following intraocular lens (IOL) implantation. The size of the posterior capsule opening was 5920 microns × 3880 microns before surgery. Intra-operatively the PCT was evident and hydro-dissection was not performed. Phaco-aspiration of the central core followed by cleaning of the cortex with the irrigation-aspiration hand-piece was carried out. The vitreous face was intact, condensed and did not prolapse into the capsular bag. The edges of the PCT were clearly visible as they were fibrosed, capsular bag fixation of the IOL could be achieved. Postoperative Scheimpflug images revealed a posterior capsule opening measuring 4840 microns × 3970 microns. His BCVA following IOL implantation improved to 20/20 in the left eye and the IOL was well-centered, in-the-bag as visualized on slit-lamp and Scheimpflug images [Fig. 3].

## Discussion

Posterior capsule tear and traumatic cataract after a non-penetrating ocular injury[1,3] and an isolated PCT are both well-recognized clinical entities.[1,3–8] The lens fibers get progressively hydrated after development of the tear resulting in cataract formation. The PCT develops thick, fibrous margins about six weeks after the trauma[4] which prevents the tear from enlarging during surgery and allows for a conventional cataract surgery to be performed. When PCT occurs, the extent of tear, amount of residual nucleus and cortex, and presence or absence of vitreous prolapse into the anterior chamber, are parameters that vary across patients.[9]

Scheimpflug imaging provides an objective way to document and quantify the tear; to quantify the density of the associated traumatic cataract if any and monitor its progression. In our case, the fact that the vitreous face was intact, there was no lens matter in the vitreous and the edges of the PCT were fibrosed allowed the surgeon to proceed with phacoemulsification. The size and shape of the PCT allowed the surgeon to assess that a posterior chamber (PC) IOL could be implanted. Additionally, the absence of any vitreous prolapse was a good prognostic indicator.

Recently, Por *et al.*,[8] suggested that blunt trauma-induced blowout PCT in children occurs due to a combination of forces: equatorial stretching pulls on the zonule and stretches the capsule and this anterior-posterior force tends to push it back thereby increasing the probability of the posterior capsule giving way. It usually occurs in young children where the lens matter is soft and elastic and the zonules are strong. The vitreous face maintains its integrity and the lens matter bulging through this tear in the posterior capsule gives an erroneous clinical profile of posterior lenticonus, a term we suggest as posterior pseudo-lenticonus.

Previously, such cases have been managed by a pars plana lensectomy.[4] Management using a clear corneal incision, phacoaspiration and PCIOL implantation in the capsular bag has also been well established now.[9]

This report highlights the use of Scheimpflug imaging in visualizing and quantifying the PCT. While slit-lamp examination does illustrate the defect, the primary advantage of the rotating Scheimpflug camera is that it allows accurate and objective quantification of the PCT. Additionally changes in the dimensions of the tear may be followed in cases where the surgeon decides to delay the surgery. The centration and tilt of the IOL can also be objectively documented following surgery. Using similar advanced imaging techniques could better help elucidate the pathogenesis of such injuries.
